# Genome-wide analysis of excretory/secretory proteins in *Echinococcus multilocularis*: insights into functional characteristics of the tapeworm secretome

**DOI:** 10.1186/s13071-015-1282-7

**Published:** 2015-12-30

**Authors:** Shuai Wang, Wei Wei, Xuepeng Cai

**Affiliations:** State Key Laboratory of Veterinary Etiological Biology, Key Laboratory of Veterinary Parasitology of Gansu Province, Lanzhou Veterinary Research Institute, Chinese Academy of Agricultural Sciences, Xujiaping 1, Yanchangbu, Lanzhou, 730046 Gansu China

**Keywords:** *Echinococcus multilocularis*, Excretory/secretory protein, Drug targets, Antigenic density

## Abstract

**Background:**

The cestode *Echinococcus multilocularis* is the causative agent of human alveolar echinococcosis (AE). However, this life-threatening disease is still difficult to treat and control, due to the lack of efficient drugs and vaccines. Excretory/secretory (ES) proteins are crucial for parasite survival and represent potential preferred targets for novel intervention strategies. However, the ES protein features in this parasite have been poorly investigated until now. The current study was carried out to identify and characterise a repertoire of ES proteins in *E. multilocularis* at the genome-wide level.

**Methods:**

Here we predicted and functionally annotated the classical and non-classical ES proteins, and comprehensively compared the features and evolution of ES and non-ES proteins in *E. multilocularis* genome using an integration of bioinformatics tools. The intervention target and antigen potentials as well as the transcription information were also investigated.

**Results:**

Computational analysis of the *E. multilocularis* proteins identified 673 putative ES proteins (6.4 %), of which 617 (91.68 %) could be supported by transcription analyses. The predicted ES proteins in *E. multilocularis* were mostly represented by molecular functions of protease inhibitors, proteases, glycoside hydrolases, immunoglobulin-like folds and growth factors. Analysis of the ratio between synonymous and non-synonymous substitution rates (dN/dS) revealed no significant difference of the evolution selection pressure on ES and non-ES protein coding genes. ES proteins showed higher antigenic density measured by AAR values as compared with the transmembrane proteins but had no significant difference of that feature with intracellular proteins. Additionally, 383 possible ideal drug targets were identified in ES proteins, of which four proteins have corresponding known drugs.

**Conclusions:**

The present study is the first to identify a repertoire of predicted ES proteins at the genome-wide level in *E. multilocularis*. The comprehensive analysis provides some novel understanding of the parasite ES protein features and a valuable resource of potential targets for future experimental studies to develop new intervention tools against this parasite.

**Electronic supplementary material:**

The online version of this article (doi:10.1186/s13071-015-1282-7) contains supplementary material, which is available to authorized users.

## Background

The tapeworm *Echinococcus multilocularis* is a cyclophyllidean cestode of great medical and agricultural importance. Its life-cycle comprises a strobilar adult stage that resides within the intestine of the definitive host (e.g. foxes and dogs), and three larval stages (oncosphere, metacestode and protoscolex) that are involved in the infection of the intermediate host (small rodents and, occasionally, humans) [1, 2]. The metacestode larvae can cause the disease alveolar echinococcosis (AE) in humans, which is one of the most dangerous helminth infections [3]. However, this deadly disease is still difficult to treat and control due to the lack of efficient drugs and vaccines [3, 4]. Among the candidate molecules that are of value to combat tapeworm infections, excretory/secretory (ES) proteins are worthy of particular attention because of their central roles in understanding host-parasite interactions [5, 6].

ES proteins of parasites are crucial for their survival inside and outside of their host organisms and can act as virulence factors or immune regulators to the host immune responses [5, 7, 8]. Therefore, they represent a preferred group of antigens for the development of new intervention strategies, such as vaccine candidates or drug targets [9–11]. Moreover, ES proteins are usually immunogenic diagnosis antigens due to their accessibility to be recognised by host immune systems and thus considerable attention has been made in ES proteins as biomarkers to detect the presence of a parasite and/or the status of the infection in different infectious diseases. ES products from *E. multilocularis* have been reported to tightly downregulate accessory cell functions of macrophages [12] and induce apoptosis and tolerogenic properties in dendritic cells, which is likely important for generating an immunosuppressive environment at infection phases [1]. The *E. multilocularis* antigens Em2 and Em492 that are involved in modulating the host-parasite interface have also been identified [6, 13, 14]. In particular, another ES protein EmTIP, an *E. multilocularis* homologue of the human T-cell immunomodulatory protein, has been shown to promote IFN-γ release by CD4+ T-cells, and is suggested as a promising lead for future studies on the development of anti-*Echinococcus* intervention strategies [1]. Recently, *E. multilocularis* have been developed as an experimental model of host-parasite interplay and parasitic immunopathology because of its advantages in culture *in vitro* and genetic manipulation under laboratory conditions, in which the excretory/secretory metabolic products are considered to play a central role [6, 15]. However, until now, the ES proteins of this tapeworm have been relatively poorly investigated.

Because experimental identification of ES proteins is time-consuming and expensive, the prediction of ES proteins from sequenced genomes is a novel alternative strategy used to prioritise the experimental study of new therapeutic and immunodiagnostic targets for human parasitic diseases [7–9, 16–19]. The availability of whole genome sequences for *E. multilocularis* [4] gives us the opportunity to systematically explore the parasite secretome using computational approaches. Here, we combined several different but highly complementary analytical approaches to predict, functionally annotate and comprehensively analyse the *E. multilocularis* secretome in detail. We believe that our genome-wide exploration of ES proteins could provide a valuable resource for future experimental studies and give a better understanding of the parasite secretome. Moreover, as *E. multilocularis* has been recently developed as an experimental model for tapeworm research, the present study will also give the clues to proteomes in other tapeworms.

## Methods

### Prediction of ES proteins of *E. multilocularis* genome

**Fig. 1:**
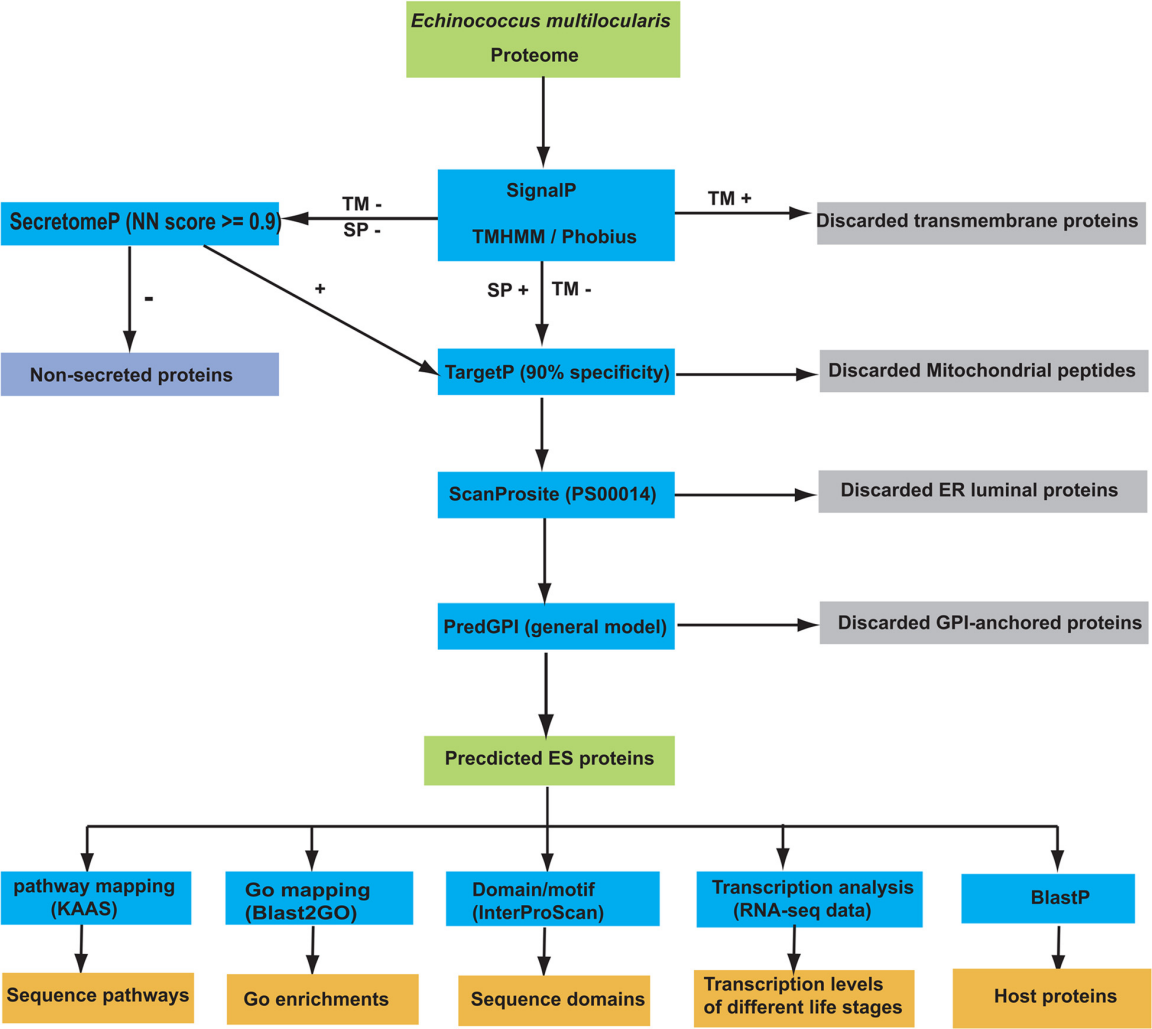
Bioinformatic workflow used for ES protein analysis. The detailed process is described in the Methods section

The proteome of *E. multilocularis* (version 3) was downloaded from GeneDB (http://www.genedb.org/Homepage). Our bioinformatics workflow is shown in Fig. 1, using a strategy of integrating several tools. The algorithm TMHMM (version 2.0) [20] was used to predict transmembrane (TM) regions. For the proteins predicted to contain only one TM domain, further TM prediction was performed by the Phobius algorithm [19] to help discriminate hydrophobic helices of TM topologies from those of signal peptides, in which only the proteins confirmed by Phobius were considered as TM proteins. All the proteins predicted to carry a TM domain were discarded for the further analysis. SignalP (version 4.1) [21] was used for predicting signal peptides of classically secreted proteins, with options of eukaryote organism categories, truncation of protein sequence at 70 amino acids and default D-cutoff values. The non-classical secreted proteins were predicted by SecretomeP (version 2.0) [22], filtered by NN-scores larger than 0.9 and other default options for mammalian organisms. All the classical and non-classical secretory proteins were merged together and the resulting list was scanned by TargetP (version 1.1) [23] to predict the subcellular localisation of mitochondrial proteins, using a specificity of 90 % and the default options for non-plant organisms. The predicted mitochondrial proteins by TargetP were discarded from the protein data set. The resulting ES proteins were subsequently scanned for the presence of ER targeting signals by PS-Scan [24] (Prosite pattern: PS00014) and GPI-anchor signals by PredGPI [25] with default parameters. For comparison, we define the proteins that are neither ES nor TM-containing proteins as “intracellular proteins” in our analysis. Therefore, the total non-ES proteins consist of the TM-containing proteins and the intracellular proteins.

### Functional annotations and expression profiling

All the predicted ES proteins were annotated for protein domains and family classifications using InterProScan (version 5) [26], including the gene ontology (GO) terms option. The KAAS server [27] was used to map ES proteins to KEGG pathways and to KEGG BRITE objects, using the single-directional best hit method to assign the orthologs (threshold of BLAST bit scores = 50). The representative gene data set for eukaryotes plus that of *Schistosoma mansoni* were used as references in KAAS mapping. Go term enrichment analysis of ES proteins was performed by BlAST2GO by Fisher’s Exact Test with Multiple Testing Correction of FDR (FDR < 0.05) (using the entire proteome as the reference group).

The transcriptomic information available in the reference [4] was used for transcription expression analysis of *E. multilocularis*. The expression levels of ES proteins were evaluated and ranked by fragments per kilobase of exon per million fragments mapped (FPKM) values based on the normalised read counts of RNA-seq Illumina reads [4]. The following life-cycle stages were involved in our analysis: metacetode, pre-gravid adult and gravid adult. To determine significant differences in the levels of gene expression between the different life-cycle stages, we defined as differentially expressed genes (DEGs) those for which the *p*-value was smaller than 0.01 and for which the fold change was larger than 2 (either up- or downregulated). For each ES protein, a relative measure of transcription in the stages was inferred by ranking individual genes from *E. multilocularis* by their FPKM values (highest to lowest). The top 25 % of genes were defined as having very high transcription, 25–50 % as high, 50–75 % as medium and 75–100 % as low. Wilcoxon Signed-Rank test, implemented in R (using the option paired = FALSE), was used to compare the expression differences between ES genes and non-ES genes.

### Analysis of dN/dS and adaptive evolution

In order to test whether the ES proteins of *E. multilocularis* have undergone higher selection pressure during evolution, we calculated the dN/dS (ω) values of the ES proteins along this parasite lineage. The whole gene sets from three tapeworms (*Echinococcus granulosus*, *E. multilocularis* and *Taenia solium*; http://www.genedb.org/) were used to identify orthologue groups by the program Inparanoid (version 4.0) [28] and Multiparanoid (version 1.0) [29]. All the transcripts that lacked intact coding regions (CDSs), that had in-frame stop codons, that had CDSs of <100 bp, or that had CDSs whose lengths were not multiples of three were discarded. To establish 1:1 orthology, each ortholog was examined for evidence of an inparalog (a paralog arising from a recent duplication) with respect to the other species. Specifically, if either gene had inparalogs, then that gene was considered recently duplicated and was excluded from the analyses of positive selection. Multiple protein-coding codon alignments were generated using ParaAT (version 1.0) [30] and Mafft (version 7.147b) [31], with all gaps in the alignments deleted. The likelihood ratio test (LRT) for selection (*P* < 0.05) on any branch of the phylogeny was performed based on the results from the Codeml program between the null hypothesis of one-ration model (model = 0) that fixed the ω =1 and the alternative hypothesis with free-ration model (model = 1) as implemented in the PAML package (version 4.7) [32]. *P*-values were computed assuming the null distribution was a 50:50 mixture of a χ^2^ (df = 2) distribution and a point mass at zero. Significance of difference between ω values (<10) of ES proteins and non-ES proteins in the free-ratio model were calculated using Wilcoxon Signed-Rank test (paired = FALSE).

### Antigenic Region abundance and drug target potential analysis

To evaluate the antigenicity potential of *E. multilocularis* secretome, the number of antigenic regions for each protein sequence was detected using the bioinformatics algorithm Bepipred [33] with a threshold of 0.90 and the method Kolaskar-Tongaonkar [34] implemented in EMBOSS packages [35] with a threshold 1.0. Only antigenic segments with length of at least 6 amino acids were included in further analyses. The Abundance of Antigenic Regions (AAR) value [36] was utilised in this study to normalise the number of antigenic regions by sequence length**.** This value was calculated as the ratio between the sequence length and the number of predicted antigenic regions for each protein. Hence, this value represents the mean number of amino acids that is needed to find one antigenic region in a protein sequence.

In order to identify the specific ES proteins in *E. multilocularis* that show no sequence similarities with its hosts, we performed homology searches by BLASTP algorithm (threshold e-value of 1e^−3^) using the entire predicted secretome as queries against the proteomes of human and dog (http://www.ensembl.org/index.html). Proteins not homologous to the host proteomes were further screened for sequence similarities against the known drug targets. Drug target sequences were extracted from the following databases: 1. ChEMBL (ftp://ftp.ebi.ac.uk/pub/databases/chembl/DrugEBIlity/releases/3.0/), 16072 drug target protein sequences and 212919 domain sequences; 2. DrugBank (http://www.drugbank.ca/), 3789 proteins; 3. Therapeutic Targets Database (http://bidd.nus.edu.sg/group/ttd/), 1973 proteins.

## Results

### Prediction of ES proteins in *E. multilocularis* genome

Of the 10,552 putative proteins in *E. multilocularis*, 2150 sequences were predicted by TMHMM to contain one or more TM regions. For the sequences (924) that were detected with only one TM domain, 784 sequences were confirmed by Phobius as transmembrane proteins, by excluding the overlapping predictions between hydrophobic regions of real TM topologies and those of signal peptides. The remaining 8542 TM-free sequences were submitted to SignalP to predict a signal cleavage site, resulting in 551 sequences (5.2 %) as classical secreted proteins. Of the 7991 sequences without a signal peptide predicted by SignalP, Secretome could classify 230 proteins (2.2 %) as non-classical secreted proteins. Combining the results above yielded a total of 781 (7.4 %) classical and non-classical proteins that were then checked by TargetP, resulting in 25 mitochondrial targeting proteins. The C-terminal [KRHQSA][DENQ]EL Prosite pattern identified 9 proteins with retention signals as ER proteins in the analysis. In addition, 74 proteins containing a GPI anchor determined by PreGPI were also discarded. In the end, a total of 673 (6.4 %) sequences (Additional file 1: Table S1) were finally predicted as ES proteins that are used for further analyses.

### Functional annotation of *E. multilocularis* secretome

Of the 673 *E. multilocularis* ES proteins, InterProScan was able to match 358 (53 %) proteins to known domains in at least one database, apart from signal peptides. The most represented InterPro terms are shown in Table 1 (complete results available from Additional file 2: Table S2). The ES proteins associated with these terms represent protein functions of peptidase inhibitors (pancreatic trypsin and cathepsin), immunoglobulin-like domains, cysteine proteases, glycoside hydrolases, taeniid antigens, cysteine-rich secretory protein family (CRISP) from CAP superfamily, homeodomain/homeobox, and growth factors (epidermal growth factor-like and EGF-like).

**Table 1:** Top 20 most represented protein domains found in ES proteins using Interproscan

InterPro IDs	Description	No. of ES (%) proteins (%)
IPR002223	Proteinase inhibitor I2, Kunitz metazoa	17 (2.53 %)
IPR013783	Immunoglobulin-like fold	15 (2.23 %)
IPR020901	Proteinase inhibitor I2, Kunitz, conserved site	15 (2.23 %)
IPR013128	Peptidase C1A	10 (1.49 %)
IPR017853	Glycoside hydrolase, superfamily	10 (1.49 %)
IPR000668	Peptidase C1A, papain C-terminal	10 (1.49 %)
IPR025660	Cysteine peptidase, histidine active site	10 (1.49 %)
IPR007110	Immunoglobulin-like domain	10 (1.49 %)
IPR000169	Cysteine peptidase, cysteine active site	10 (1.49 %)
IPR003599	Immunoglobulin subtype	9 (1.34 %)
IPR008860	Taeniid antigen	9 (1.34 %)
IPR014044	Cysteine-rich secretory protein family (CAP)	9 (1.34 %)
IPR013032	EGF-like, conserved site	9 (1.34 %)
IPR025661	Cysteine peptidase, asparagine active site	9 (1.34 %)
IPR000742	Epidermal growth factor-like domain	9 (1.34 %)
IPR013201	Proteinase inhibitor I29, cathepsin propeptide	8 (1.19 %)
IPR009057	Homeodomain-like	8 (1.19 %)
IPR013098	Immunoglobulin I-set	7 (1.04 %)
IPR001356	Homeobox domain	6 (0.89 %)
IPR007087	Zinc finger, C2H2	6 (0.89 %)

**Fig. 2:**
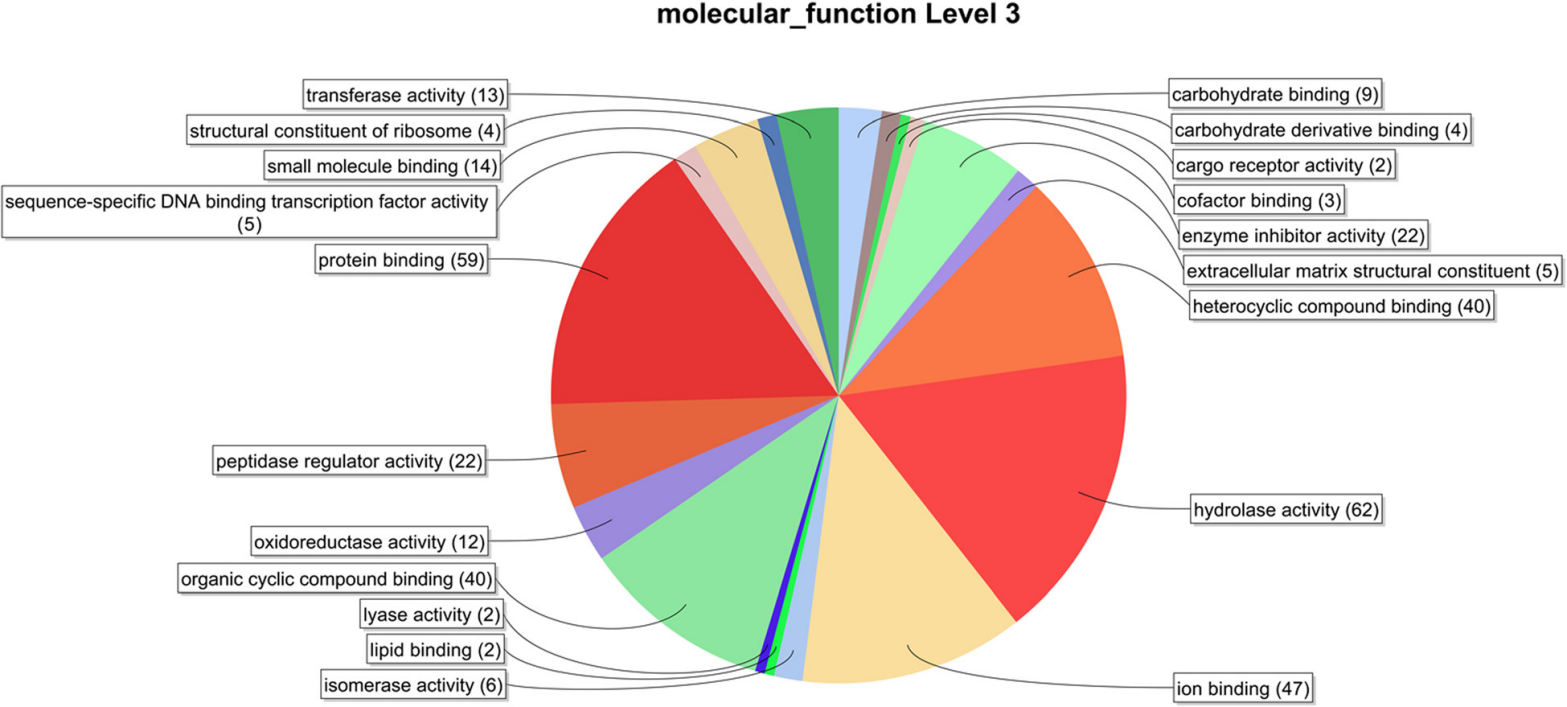
Molecular function ontology distribution of *E. multilocularis* predicted ES proteins on third level subcategory

In total, 245 ES proteins were assigned to 535 GO terms (Additional file 1: Table S1), which could be divided into 162 GO terms originating from the Biological process (Additional file 3: Figure S1), 67 GO terms from the Cellular component (Additional file 4: Figure S2) and 306 GO terms from the Molecular function (Fig. 2 and Additional file 5: Figure S3). A summary of GO term annotations at a third level subcategory from the Molecular function is provided in Fig. 2 (a summary of a second level subcategory in Additional file 5: Figure S3). Of the Molecular function at a second level (Additional file 3: Figure S1), the binding, catalytic activity and enzyme regulator activity terms represented with 129 annotations (49 %), 95 annotations (36 %) and 23 annotations (9 %) respectively, almost accounted for 100 % of all the annotations. The parental term binding (at the second level) includes the third level subcategory terms protein binding (59 annotations), ion binding (47), heterocyclic compound binding (40), organic cyclic compound binding (40), carbohydrate binding (9), small molecule binding (14), cofactor binding (3), lipid binding (2), and carbohydrate derivative binding (4). The second largest second-level term catalytic activity was represented by the third-level terms hydrolase activity (62), oxidoreductase activity (12), isomerase activity (6), transferase activity (13) and lyase activity (2). The parental second-level term enzyme regulator activity includes the third-level terms peptidase regulator activity (23) and metalloenzyme regulator activity (1).

**Fig. 3:**
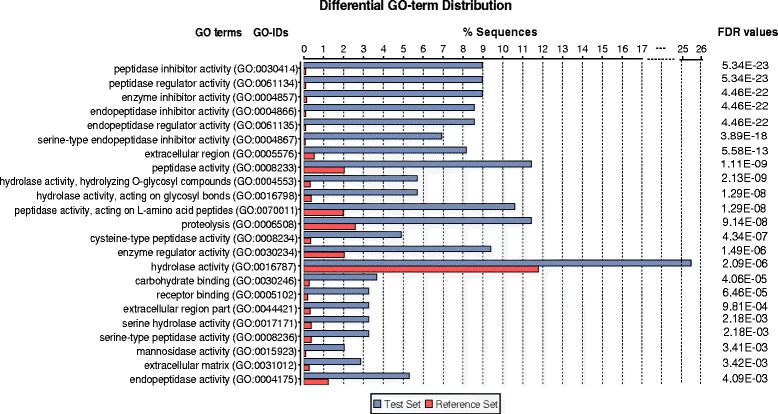
Differential GO term distribution between the predicted secretome and the whole proteome of *E. multilocularis*. The test set is the predicted secretome of *E. multilocularis*; the reference data set is the whole proteome of *E. multilocularis*. The FDR values represent the FDR values in the enrichment test

The significantly enriched terms filtered by Fisher’s Exact Test (FDR < 0.01) in the *E. multilocularis* secretome, are shown in Fig. 3. The terms mostly related to peptidase inhibitor activity, peptidase activity, hydrolase activity, carbohydrate binding and receptor binding are significantly enriched in the Molecular Function category. Of the terms associated to peptidases, the serine-type peptidase activity and cysteine-type peptidase activity are significantly enriched in the ES proteins. Additionally, several other enriched terms representing hydrolase activity were also detected, including hydrolase activity acting on glycosyl bonds and on L-amino acid peptides, mannosidase activity and alpha-mannosidase activity. For the Biological process category, the most enrichment terms are: proteolysis, single-multicellular organism process, multicellular organismal process, and multicellular organismal development. The terms extracellular region and extracellular matrix show enrichment in the Cellular component category.

KEGG pathway analysis by KAAS assigned 187 ES proteins into 135 pathways (Additional file 1: Table S1). The top 10 pathways are listed in Table 2 (the complete data set is in Additional file 6: Table S3). The most represented pathway is the lysosome, followed by the protein processing in endoplasmic reticulum pathway. Several other pathways, such as signaling pathways regulating pluripotency of stem cells, hippo signaling pathway, PI3K-Akt signaling pathway, focal adhesion and glycan degradation were also involved in the top pathways. In particular, some signaling pathways contained assigned components related to growth factors, like insulin-like growth factor (IGF), wingless-type MMTV integration site family member 1 (Wnt1) and EGF, could also be found in the KEGG pathway analysis.

**Table 2:** Top 10 most represented KEGG pathways found in ES proteins predicted by KAAS

Pathway name	No. of ES proteins represented (%)
Lysosome	18 (2.7 %)
Protein processing in endoplasmic reticulum	14 (2.1 %)
Pathways in cancer	7 (1.1 %)
Signaling pathways regulating pluripotency of stem cells	6 (0.9 %)
Hippo signaling pathway	6 (0.9 %)
PI3K-Akt signaling pathway	6 (0.9 %)
Focal adhesion	6 (0.9 %)
Proteoglycans in cancer	5 (0.7 %)
Hedgehog signaling pathway	5 (0.7 %)
Other glycan degradation	5 (0.7 %)

### Transcription profiling analysis

**Fig. 4:**
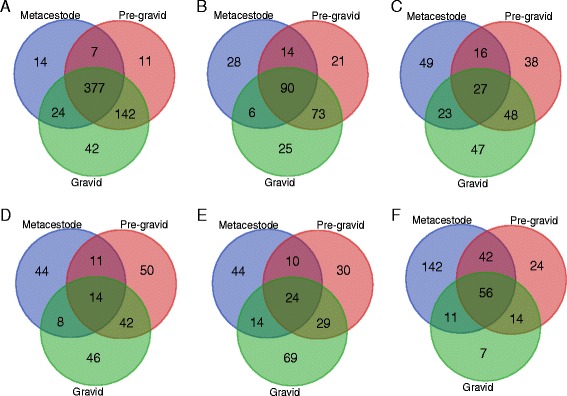
Venn diagrams of transcription analysis of the predicted ES proteins in the three development stages (metacestode, pre-gravid and gravid adult) of *E. multilocularis*. The venn diagrams show the gene numbers of the ES protein coding genes that are supported by transcription (**a**), expressed at a very high expression level (top 25 %) (**b**), expressed at a high expression level (top 25–50 %) (**c**), expressed at a medium expression level (top 50–75 %) (**d**), expressed at a low expression level (top 75–100 %) (**e**), and not expressed (**f**), respectively

**Fig. 5:**
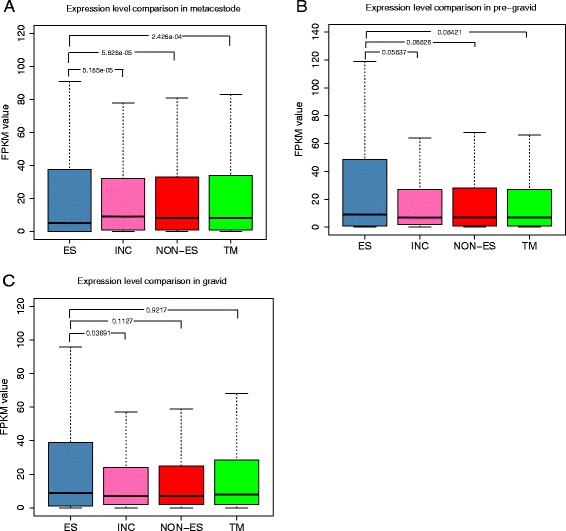
Expression level comparisons between ES protein and non-ES protein coding genes. *Abbreviations*: ES, ES protein coding genes; INC, proteins that are not ES or TM-containing protein coding genes; TM, TM containing protein coding genes; NON-ES, total non-ES protein (consisting of INC and TM proteins) coding genes; *P*-values calculated using Wilcoxon Signed-Rank test for comparisons between different data sets are displayed. **a** Expression level comparisons in the metacestode stage; **b** Expression level comparisons in the pregravid adult; **c** Expression level comparisons in the gravid adult

According to the transcription analysis using RNA-seq data, 91.68 % (617) of the 673 ES proteins in *E. multilocularis* could be detected to have transcript support in at least one life-cycle stage. Specifically, for the ES protein coding genes that could be supported by RNA transcripts, 377 genes were shared by all the three stages, whereas 14, 11 and 42 genes were observed as stage-specific genes in metacestode, pre-gravid and gravid stages, respectively (Fig. 4). Otherwise, 138, 198 and 194 ES proteins were expressed at a very high level (top 25 %) in the three stages accordingly (Fig. 4 and Additional file 7: Table S4), of which 90 genes have very high expression levels among all the three stages. For the 90 very highly expressed genes, 51 genes could be annotated by InterproScan, in which the most represented domains were related to peptidase inhibitor activity, ion binding, peptidase activity, hydrolase activity and Taeniidae antigen. Moreover, 112 genes have significantly different expressions among the three stages (Additional file 7: Table S4). Out of the 383 ES genes specific in *E. multilocularis*, which exhibited no similarities to proteomes of dog and human (threshold e-value of 1e^−3^), 348 had RNA transcripts in at least one stage (Additional file 8: Figure S4). Moreover, 92, 131 and 116 *E. multilocularis*-specific genes were detected with very high expression levels in the metacestode, pre-gravid and gravid, stages respectively. Of these highly expressed ES genes, 48 genes were expressed in all the three stages (Additional file 7: Table S4 and Additional file 8: Figure S4). Comparisons of FPKM values between ES protein coding genes and each group of non-ES protein coding genes (i.e. the total non-ES, TM-containing and intracellular protein coding genes) revealed that there were no significant differences (*p*-value <0.01) of expression levels between them in the adult life stage, respectively (Fig. 5). Interestingly, lower expression level distribution of ES protein coding genes were supported by the Wilcoxon Signed-Rank tests, compared with those of the total non-ES (*p*-value = 5.628e^−05^), TM-containing (*p*-value = 2.426e^−04^), and intracellular protein coding genes (*p*-value = 5.185e^−05^) in the metacestode stage, respectively (Fig. 5).

### Analysis of dN/dS and adaptive evolution

**Fig. 6:**
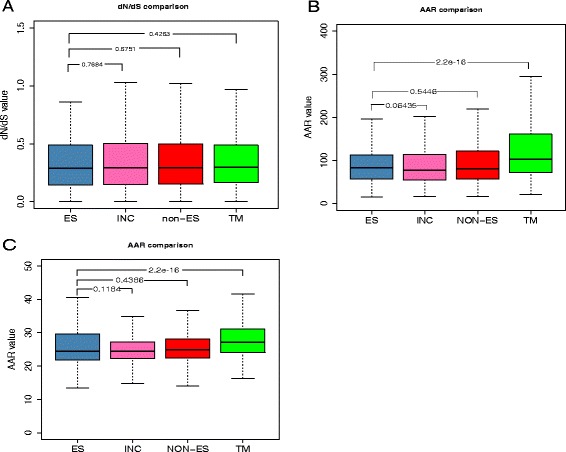
Comparisons of dN/dS and AAR values for ES and non-ES protein coding genes. Abbreviations as in Fig. 5. *P*-values calculated using Wilcoxon Signed-Rank test for comparisons between different data sets are displayed. **a** dN/dS value comparison between ES protein (*n* = 263) and non-ES proteins (INC , *n* = 4,325; NON-ES, *n* = 5,535; TM, *n* = 1,210); **b** AAR value comparisons based on the method Bepipred (ES, *n* = 673; INC, *n* = 7,869; NON-ES, 9,879; TM, n = 2,010); **c** AAR value comparisons based on the method Kolaskar-Tongaonkar (ES, *n* = 673; INC, *n* = 7,869; NON-ES, *n* = 9,879; TM, *n* = 2,010)

We determined 6105 1:1 orthology groups among *E. multilocularis*, *E. granulosus* and *T. solium*. Using the free-ratio model, we calculated the ω value of each gene along the lineage *E. multilocularis*. The mean ω values (with all the values >10 excluded) for all the genes (*n* = 5,798), ES protein coding genes (*n* = 263), and non-ES protein coding genes (*n* = 5,535) were 0.4470, 0.5477 and 0.4423 respectively, suggesting strong purifying selection on each group in *E. multilocularis*. Interestingly, by comparing the ω value distributions of the ES and non-ES proteins, we found no significant differences of evolutionary selection pressure in average among these groups in *E. multilocularis* (Fig. 6a). In total, 252 genes were identified as positively selected genes (PSGs) determined by the LRT tests (*p*-value <0.01), of which only 11 genes encoded for the ES proteins (Additional file 9: Table S5). All these PSGs were supported by transcription data, in which the very highly expressed genes protease inhibitor I25 (cystatin) and EG19 antigen were under positive selection.

### Antigenic region and drug target potential analyses

The AAR value [36] was used to define the mean number of amino acids between antigenic regions per sequence. Hence, a lower AAR value means that a protein has a higher epitope density. We determined the AAR values for the 673 ES proteins, which were 95.77 on average for the BepiPred method and 28.05 for the Kolaskar-Tongaonkar method, while TM-domain containing proteins (*n* = 2,010) had significantly higher AAR value with average one antigenic region each 133.33 amino acids for the BepiPred method and 28.96 amino acids for the Kolaskar-Tongaonkar method (Table 3). However, no statistically significant differences of AAR value distributions between ES proteins and intracellular proteins (*n* = 7,869; 96.36 for the BepiPred method and 28.53 amino acids for the Kolaskar-Tongaonkar method) were revealed by the Wilcoxon Signed-Rank test (*p*-value < 0.01) (Fig. 6b–c). Hence in our analysis, although the epitope density in ES proteins of *E. multilocularis* is slightly lower than for total non-ES proteins (*n* = 9,879; 103.54 and 28.62), the real difference resulted from the higher AAR values of TM domain-containing proteins.

**Table 3:** Abundance of antigenic regions (AAR) for different *E. multilocularis* protein datasets

Protein dataset	No. of proteins in the dataset	Average of AAR values (BepiPred)	Average of AAR values (Kolaskar)
ES proteins	673	95.77	28.05
TM-containing proteins	2010	133.33	28.96
Intracellular proteins	7869	96.36	28.53
Non-ES proteins	9879	103.54	28.62

BLASTP homology search (threshold e-value of 1e^−3^) of the 673 predicted ES proteins from *E. multilocularis* revealed 284 matches within the human proteome, and 287 matches within the dog proteome. Consequently, we found 389 ES and 386 ES proteins had no sequence similarity against human and dog proteins respectively (threshold e-value of 1e^−3^). Combing the two datasets, 383 proteins were found specific to the parasite against the proteomes of both human and dog (Additional file 1: Table S1). These parasite-specific proteins were further searched for sequence similarity against known drug targets available from the three drug databases (see methods). Of the 383 predicted ES proteins, only four *E. multilocularis* ES proteins (EmuJ_000059500.1, EmuJ_000879900.1, EmuJ_000381000.1, EmuJ_001070600.1) were found to exhibit similarities with 28 known drug targets, homologous to glycoside hydrolase, beta-D-xylosidase 2, low-density lipoprotein receptor and neuropeptide F, respectively.

## Discussion

The availability of genomic and transcriptomic data sets for *E. multilocularis* provides unprecedented opportunities to explore ES proteins that are essential for the survival of this parasite. In the current study, a repertoire of 673 ES proteins in *E. multilocularis* were identified and annotated by a pipeline established on the combination of multiple bioinformatics approaches. These ES proteins represents 6.4 % of the entire proteome, comparable to those reported in other parasites [7–9, 36]. Most of these ES proteins (91.68 %) can be supported by RNA transcription, which confirms their potential participation in the parasite life. In particular, the ES genes expressed in the metacestode stage (422), which directly interfaces with host tissues, possibly has more potential to function as key players in the host-parasite interactions [6].

Functional information of the ES proteins was obtained through the GO term, domain and pathway analyses. Similar to the case in the *T. solium* secretome [36], a relatively large part of the domains were related to peptidase inhibitors and peptidases that were significantly enriched in the *E. multilocularis* secretome (Figs. 2 and 3). Secreted proteases have been reported to be key in host-tissue degradation, excystment/encystment, tissue invasion, and larval migration for a range of helminths [37, 38], including tapeworms [39]. In addition, they are involved in modulating host immune responses against parasitic helminths [5, 6, 40]. For the protease inhibitors, they probably have capacity to regulate host protease activities and host immune responses for survival [41–43]. Therefore, it is not surprising to identify a high proportion of peptidases and peptidase regulating proteins in the secretomes of *E. multilocularis* and *T. solium*. Moreover, as peptidases are unusually immunogenic, these secreted proteases may have the potential to be exploited as ideal serodiagnostic markers and vaccine targets. The enrichment of peptidase-related proteins in the two parasite secretomes suggested that these proteins might play critical roles in survival and interactions with hosts for taeniid tapeworms. Of the most represented domains in the *E. multilocularis* secretome, ES proteins with immunoglobulin-like domains, CAP domains, taeniid antigen domains and of biological activities that are strongly related to the typical functions of secreted proteins, are shared with those of the *T. solium* secretome. In particular, ES proteins from Venom Allergen-Like family were found, which belongs to the cysteine-rich secretory protein family (CAP domain). Members from this family have been identified from the ES products of several trematode species and were suggested as potential modulators of host immune function and components of sexual development during the infection processes [44–48]. We also found several glycoside hydrolases among the top InterPro terms, all of which could be detected with transcript expressions in all of the three life-cycle stages (Additional file 7: Table S4). Proteomic analysis of the ES products from larval stages of *Ascaris suum* also revealed high abundance of glycosyl hydrolases [49]. This could suggest that the degradation of complex carbohydrates may form an essential part of the energy metabolism of these parasitic helminths once they establish in the intestine of the definitive host or tissues of the intermediate host. Along with the well-known ES proteins, about half of the predicted ES proteins (47 %) could not be assigned to any known domain, whereas most of them could be supported by RNA transcription levels (Additional file 7: Table S4). Further investigations of these molecules might lead to new and innovative approaches for the treatment and control of these parasitic diseases.

KEGG pathway mapping analysis predicted ES proteins to be most frequently located in the lysosome (Table 2), similar to the case in the *T. solium* [36] and *Dermanyssus gallinae* secretomes [7]. This phenomenon might result from the large number of lysosome-related proteins such as proteases, lipases and glycosyl hydrolases which may act as hydrolases in the lysosome [50]. These proteins act as hydrolases in the lysosome and may be involved in the degradation of host in the tapeworms**.** Other most representatively mapped KEGG pathways, such as signaling pathways regulating pluripotency of stem cells, pathways in cancer and the hippo signaling pathway were mostly related to some ligands in the pathways, involving von Willebrand factor (VWF), EGF, IGF, bone morphogenetic protein 2/4 (BMP4), and wingless-type MMTV integration site family member 1 (Wnt1). These growth factors and their pathways have extensive functions on growth, metabolism and development during parasite life-cycle [2]. In addition, we also identified nine taeniid antigens (Antigen B) that are found mostly in taeniid cestodes, from the most represented ES proteins. In fact, this protein family was also identified in the *T. solium* secretome [36] and has been well studied for its highly immunogenic properties that can be recognised by more than 80 % of sera from patients with AE [50]. Nevertheless, its precise biological function remains undetermined in these tapeworms.

Investigating the number of synonymous and non-synonymous substitutions along a lineage can provide information about the degree of selection for a species during evolution. Although the ES proteins were directly recognised by host immune systems in the host-parasite interaction, no significant difference of evolution selection pressure between ES and non-ES proteins coding genes was detected along *E. multilocularis* lineage in our analysis. This implies that host immune system might make relatively limited selection effects on the ES protein evolution during the evolution of this parasite after divergence from the common ancestor with *E. granulosus.* This is consistent with the lower ratio of PSGs in ES protein coding genes. Interestingly, the protease inhibitor cystatin and EG19 antigen were under positive selection determined by the LRT test. These two genes were both among the very highly expressed genes in the metacestode life-cycle stage. Recently, some studies have reported that nematode cystatins can regulate host protease activity and modulate host immune responses [39, 40]. Although the biological roles of EG19 have been poorly investigated, the present result implies that this protein might possess important functions for environment adaptation of this parasite.

High epitope density in a single protein molecule has been suggested to significantly enhance their antigenicity and immunogenicity [36, 51]. Calculation of AAR value of a protein is an effective way to normalise the antigenic region numbers by the sequence length in a protein [36]. In our analysis, we observed the average AAR values for ES proteins in the *E. multilocularis* genome were ~95.77 for the Bepipred method and ~28.05 for the Kolaskar-Tongaonkar method, i.e. values very similar to those of the predicted *T. solium* secretome (93.6 for the Bepipred method; 26.2 for the Kolaskar-Tongaonkar method), the known *E. multilocularis* ES proteins (92.0; 28.0) and other helminth secretomes (65.9–105.0; 24.6–29.1) determined by Gomez S and colleagues [36]. Additionally, statistical difference of AAR value distributions between ES proteins and TM-containing proteins was detected for both methods, implying distinguishable immunological features between them. However, the Abundance of Antigenic Regions (AAR) for the *E. multilocularis* secretome is not significantly enriched with antigenic regions as compared to the intracellular proteins in our analysis. This implies that the low AAR value might not be a typically unique feature for ES proteins in *E. multilocularis*. However, due to the accessibility to be recognised by the host immune system, the higher epitode density of the ES proteins as compared with that of transmembrane proteins can probably make the ES proteins perfect antigens to capture antibodies from infected patients or animals. We found that 383 ES proteins identified in *E. multilocularis* had no sequence similarity against human and dog proteins. Because of the absence in their hosts, these ES proteins would be preferred biological markers and the antibodies can be used to directly detect the ES antigens in infected hosts through a sandwich ELISA. Moreover, given the key roles of the secretome in parasite survival, efficient drugs with mild side effects may be developed on the basis of these parasite-specific ES proteins. Particularly, the ES proteins with very high expression levels involved in both the metacestode and gravid adult stages may provide more useful opportunities to find new interventions against both *E. multilocularis* larvae and adults. Of the predicted specific ES proteins, homologues to known drug targets could be found, implying the possibility that useful drugs against this parasite can be found by screening known drugs or chemicals. Among these known potential targets, Neuropeptide F which is originally isolated from the flatworm *Moniezia expansa* [52, 53] has been considered as a promising target for novel anthelmintics, due to its widespread expression in flatworm parasites [53, 54].

## Conclusions

This study applied an integrated pipeline to identify and comprehensively characterise the *E. multilocularis* ES proteins at the genome-wide level for the first time. Novel insights about the functions, antigenic density and adaptive evolution of these proteins were discovered. Additionally, it provides a valuable resource of proteins, which constitute promising candidates for drug, diagnosis or vaccine targets for further experimental research that may lead to new intervention strategies against this parasite. Nevertheless, bioinformatic analyses *in silico* are highly algorithm-dependent to identify sequence features and thus future experimental studies on the proteomic level are necessary to confirm and improve the predicted secretome.

## Additional files

Additional file 1: Table S1. Comprehensive annotation information for all the predicted ES proteins in *E. multilocularis*. (XLS 116 kb) Additional file 2: Table S2. The complete domain annotations of all predicted ES proteins in *E. multilocularis* using InterProScan. (XLS 67 kb) Additional file 3: Figure S1. Biological process ontology distribution of *E. multilocularis* predicted ES proteins on third level subcategory. (PDF 4 kb) Additional file 4: Figure S2. Cellular component ontology distribution of *E. multilocularis* predicted ES proteins on third level subcategory. (PDF 2 kb) Additional file 5: Figure S3. Molecular function ontology distribution of *E. multilocularis* predicted ES proteins on second level subcategory. (PDF 2 kb) Additional file 6: Table S3. The KEGG pathways found in *E. multilocularis* predicted ES proteins by KAAS. (XLS 24 kb) Additional file 7: Table S4. Transcription analysis of all predicted ES proteins in *E. multilocularis*. (XLS 94 kb) Additional file 8: Figure S4. Venn diagrams of expression levels of ES protein coding genes in different developments. The venn diagrams are displayed for: the genes that have expressions (A), the genes with very high expression levels (B), the genes with high expression levels (C), the genes with medium expression levels (D), the genes with low expression levels (E), and the genes without expressions (F). (PDF 285 kb) Additional file 9: Table S5. Positively selected genes of ES protein coding genes in *E. multilocularis*. (XLS 14 kb)

## Abbreviations

ES: Excretory/Secretory
INC: Intracellular
AE: Alveolar echinococcosis
TM: Transmembrane
FPKM: fragments per kilobase of exon per million fragments mapped
ER: Endoplasmic reticulum
CRISP: Cysteine-rich secretory protein family
VWF: Willebrand factor
EGF: Epidermal growth factor
IGF: Insulin-like growth factor, BMP4, bone morphogenetic protein 2/4
Wnt1: Wingless-type MMTV integration site family member 1
