# Supplementary figures and images for: Genome-wide analysis of excretory/secretory proteins in Echinococcus multilocularis: insights into functional characteristics of the tapeworm secretome
Source: Parasit Vectors. 2015 Dec 30;8:666. doi: 10.1186/s13071-015-1282-7 (PMC4696181; doi:10.1186/s13071-015-1282-7)

# biological\_process Level 3

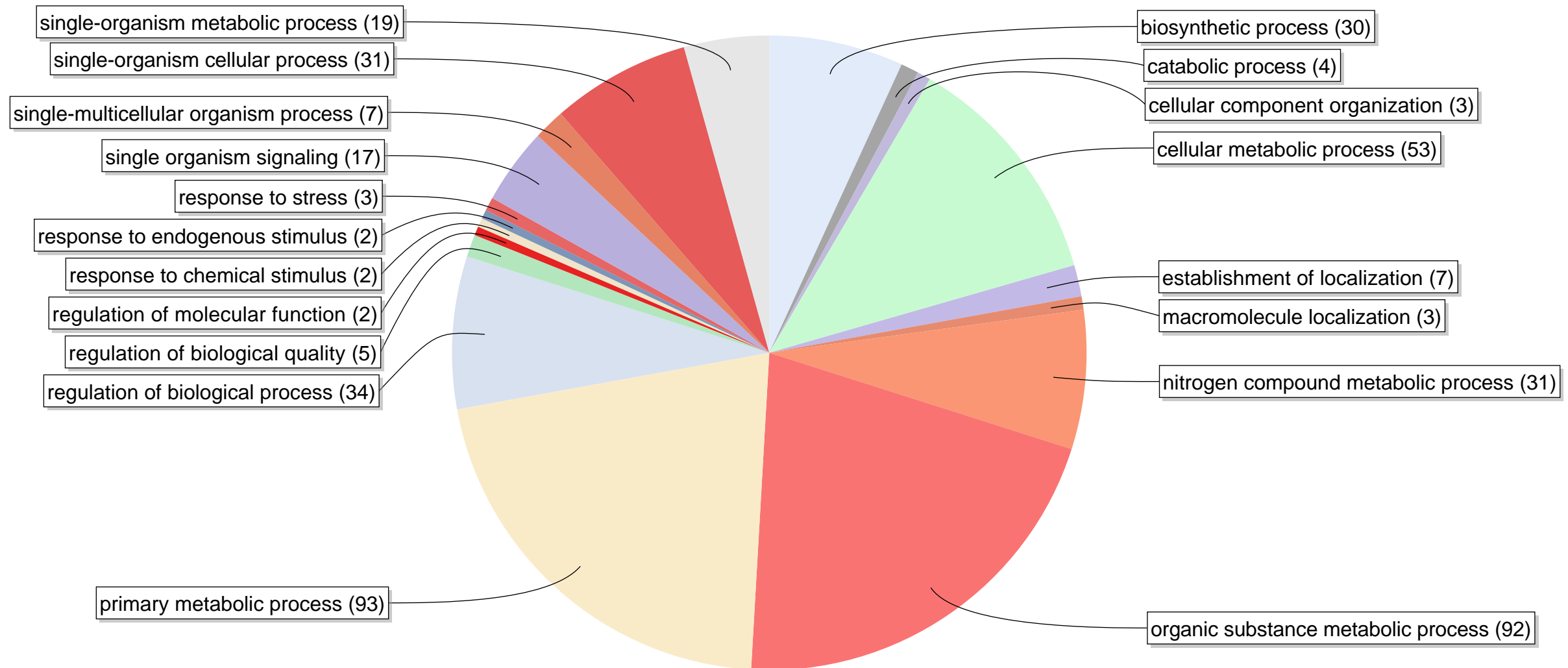

Supplement: Additional file 3: Figure S1. — Biological process ontology distribution of E. multilocularis predicted ES proteins on third level subcategory. (PDF 4 kb) [file 13071_2015_1282_MOESM3_ESM.pdf]

**cellular\_component Level 3**

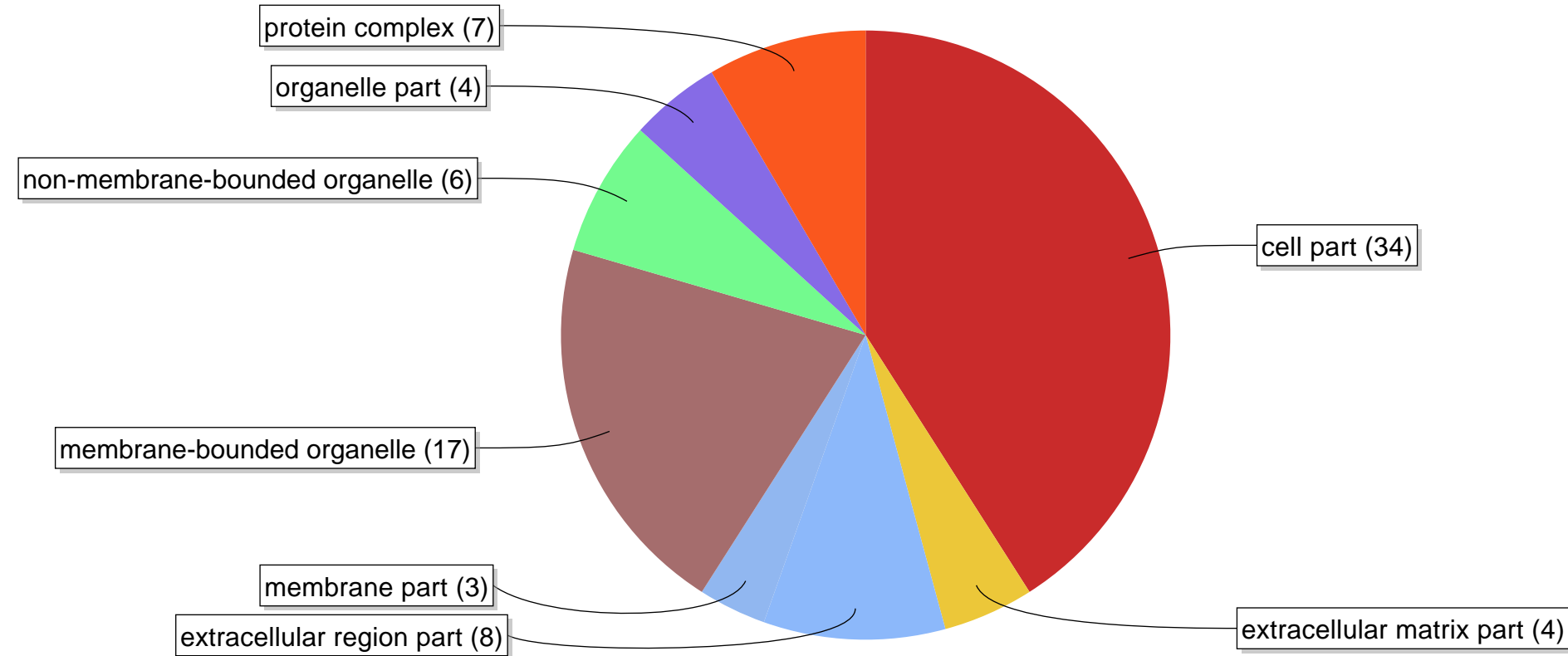

Supplement: Additional file 4: Figure S2. — Cellular component ontology distribution of E. multilocularis predicted ES proteins on third level subcategory. (PDF 2 kb) [file 13071_2015_1282_MOESM4_ESM.pdf]

## molecular\_function Level 2

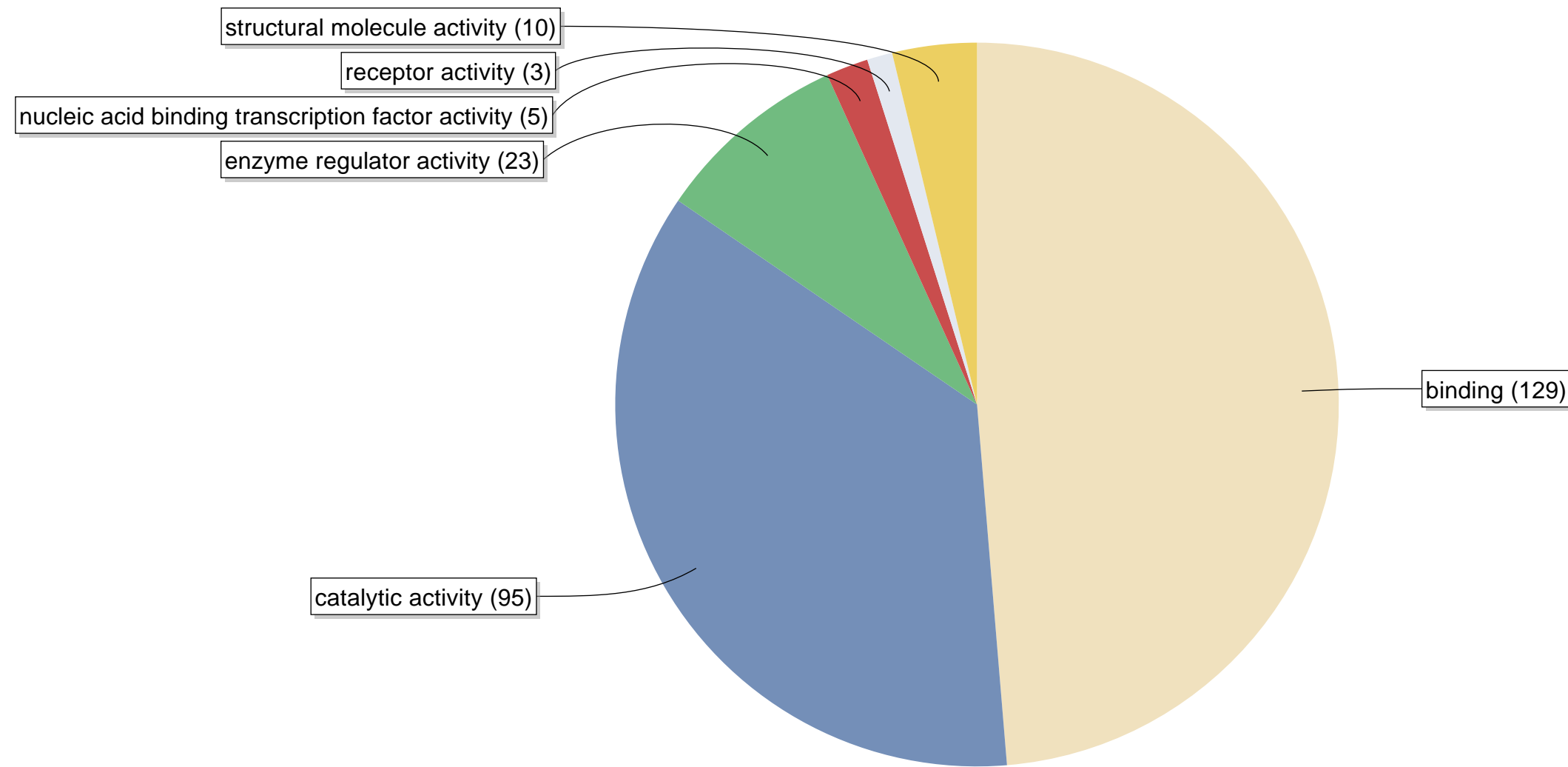

Supplement: Additional file 5: Figure S3. — Molecular function ontology distribution of E. multilocularis predicted ES proteins on second level subcategory. (PDF 2 kb) [file 13071_2015_1282_MOESM5_ESM.pdf]

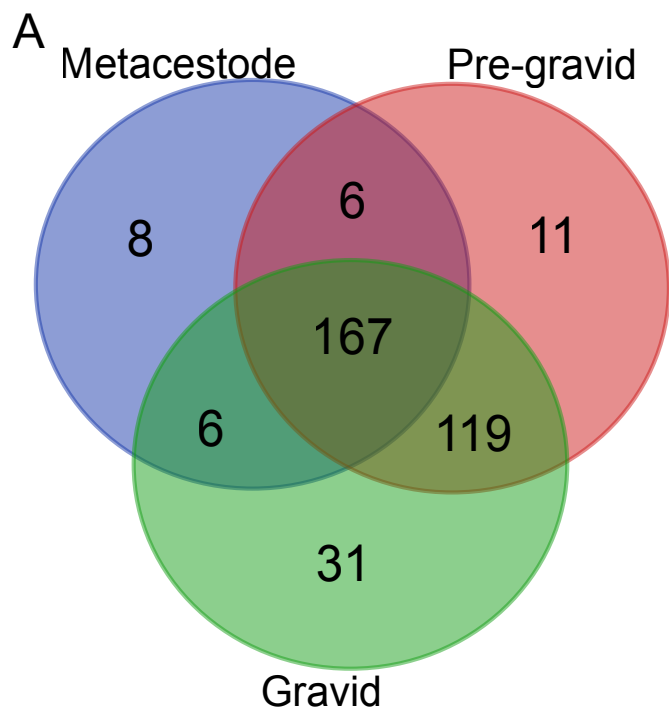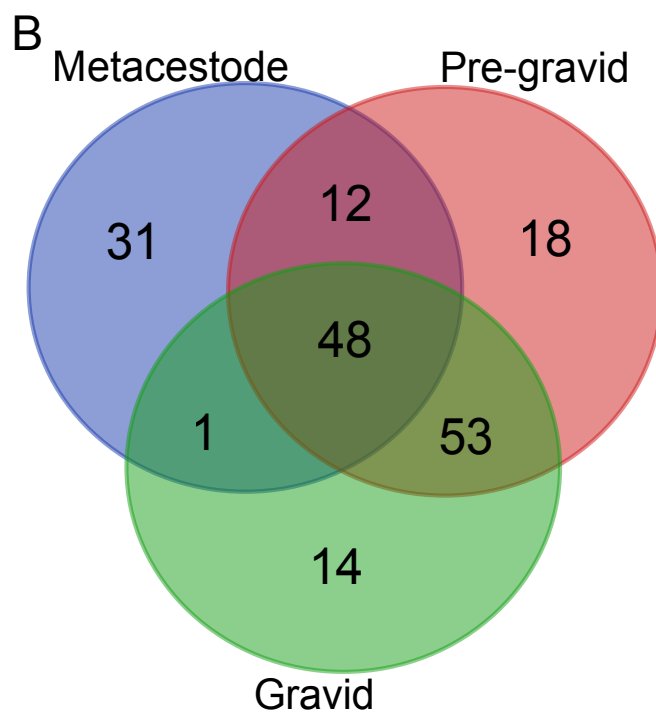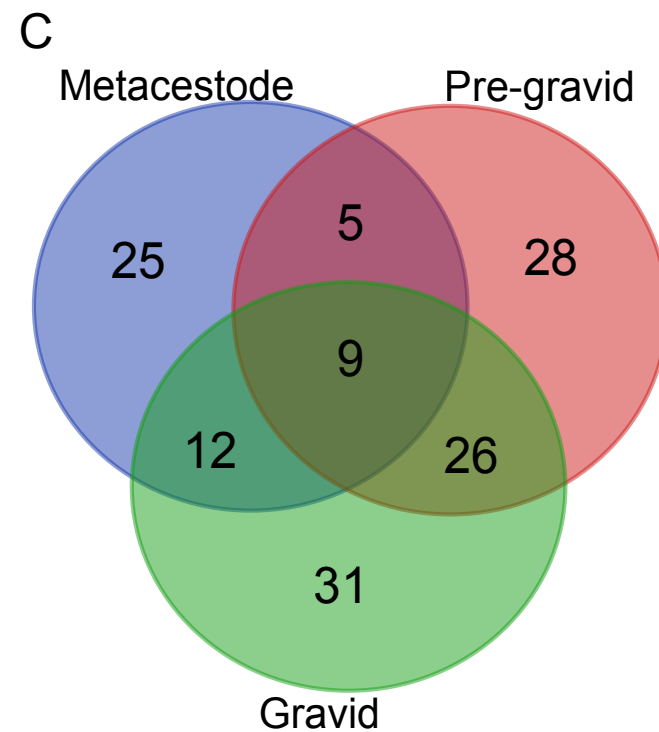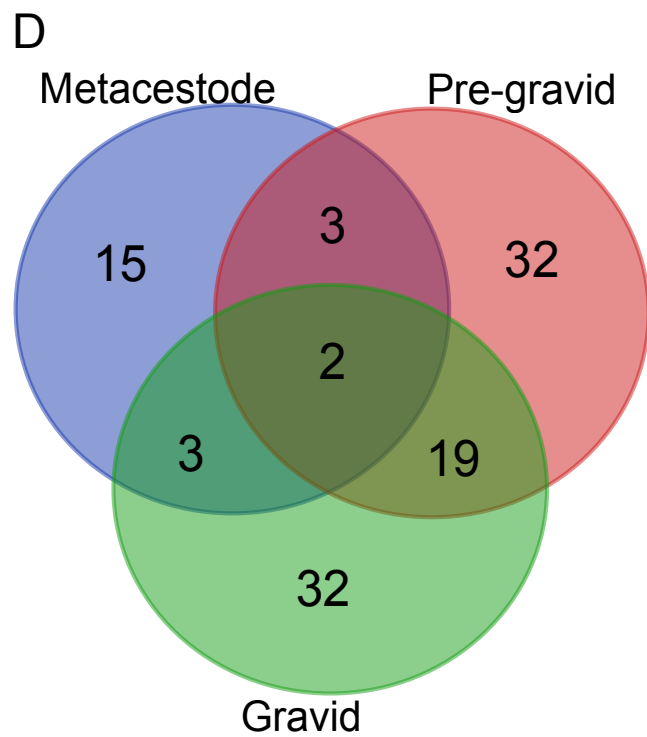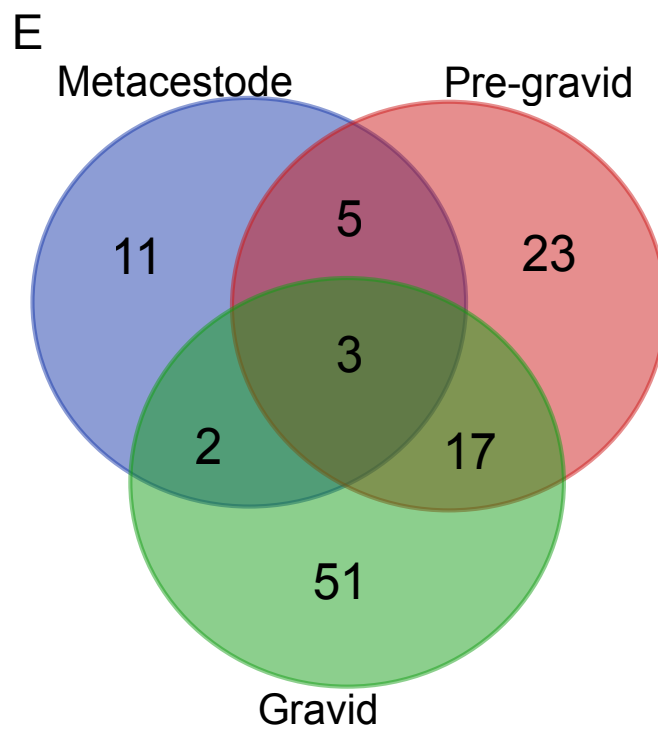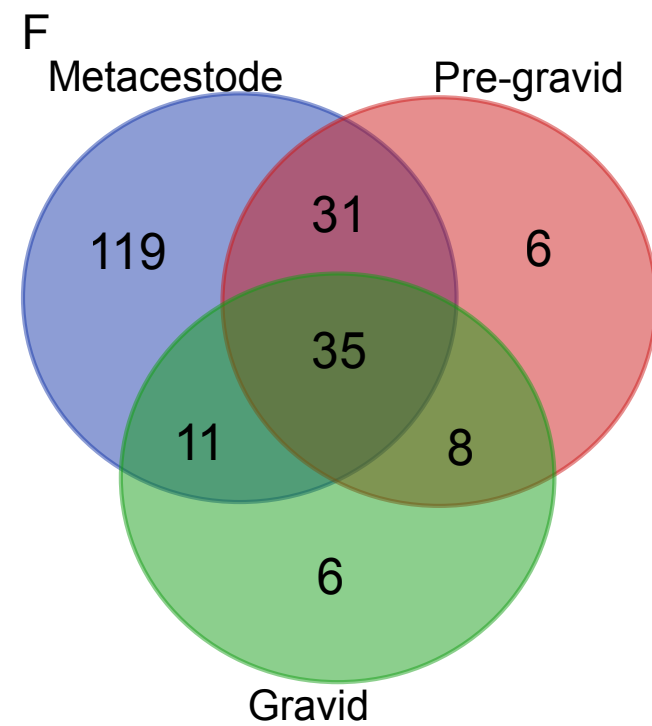

Supplement: Additional file 8: Figure S4. — Venn diagrams of expression levels of ES protein coding genes in different developments. The venn diagrams are displayed for: the genes that have expressions (A), the genes with very high expression levels (B), the genes with high expression levels (C), the genes with medium expression levels (D), the genes with low expression levels (E), and the genes without expressions (F). (PDF 285 kb) [file 13071_2015_1282_MOESM8_ESM.pdf]
